# Connecting Social Psychology and Deep Reinforcement Learning: A Probabilistic Predictor on the Intention to Do Home-Based Physical Activity After Message Exposure

**DOI:** 10.3389/fpsyg.2021.696770

**Published:** 2021-07-12

**Authors:** Patrizia Catellani, Valentina Carfora, Marco Piastra

**Affiliations:** ^1^Department of Psychology, Catholic University of Milan, Milan, Italy; ^2^Department of Industrial, Computer and Biomedical Engineering, University of Pavia, Pavia, Italy

**Keywords:** probabilistic predictor, Dynamic Bayesian Network, message framing, home-based physical activity, intention change

## Abstract

Previous research has shown that sending personalized messages consistent with the recipient's psychological profile is essential to activate the change toward a healthy lifestyle. In this paper we present an example of how artificial intelligence can support psychology in this process, illustrating the development of a probabilistic predictor in the form of a Dynamic Bayesian Network (DBN). The predictor regards the change in the intention to do home-based physical activity after message exposure. The data used to construct the predictor are those of a study on the effects of framing in communication to promote physical activity at home during the Covid-19 lockdown. The theoretical reference is that of psychosocial research on the effects of framing, according to which similar communicative contents formulated in different ways can be differently effective depending on the characteristics of the recipient. Study participants completed a first questionnaire aimed at measuring the psychosocial dimensions involved in doing physical activity at home. Next, they read recommendation messages formulated with one of four different frames (gain, non-loss, non-gain, and loss). Finally, they completed a second questionnaire measuring their perception of the messages and again the intention to exercise at home. The collected data were analyzed to elicit a DBN, i.e., a probabilistic structure representing the interrelationships between all the dimensions considered in the study. The adopted procedure was aimed to achieve a good balance between explainability and predictivity. The elicited DBN was found to be consistent with the psychosocial theories assumed as reference and able to predict the effectiveness of the different messages starting from the relevant psychosocial dimensions of the recipients. In the next steps of our project, the DBN will form the basis for the training of a Deep Reinforcement Learning (DRL) system for the synthesis of automatic interaction strategies. In turn, the DRL system will train a Deep Neural Network (DNN) that will guide the online interaction process. The discussion focuses on the advantages of the proposed procedure in terms of interpretability and effectiveness.

## Introduction

Doing physical activity is essential for people's health and well-being (Hyde et al., 2013; Rhodes et al., 2017). During the lockdown due to the COVID-19 pandemic, this role of physical activity has become even more crucial and an increase in physical activity at home has become essential to keep in exercise despite the constraints of external mobility (Taylor et al., 2020; University of Virginia Health System, 2020). Even when we are aware of the benefits associated with physical activity, this awareness does not necessarily translate into consistent behavior. This is because the psychological factors related to physical activity are many and their relationships are complex. Understanding these relationships is essential to develop personalized and effective intervention strategies, which can be addressed to as many people as possible and be economically sustainable.

Some previous research has investigated how to promote physical activity using automatic interaction systems, such as artificial intelligence chatbot or personalized physical activity coaching based on machine learning (Dijkhuis et al., 2018; Aldenaini et al., 2020; Zhang et al., 2020). However, a full understanding of the theoretical guidance and practices on designing automatic interaction systems to support the increase in people's physical activity is still lacking (Zhang et al., 2020). Such understanding should include the development of empirically testable theoretical models, which consider the psychosocial processes related to behavior planning and how communication can influence it.

In the present study, we developed an empirically testable model to facilitate the promotion of physical activity thanks to the application of artificial intelligence. To do so, we first collected data on a sample of participants exposed to different messages promoting home-based physical activity during the first lockdown due to the Covid-19 epidemic in 2020. Participants were involved in an experimental procedure articulated in three phases: (a) filling out a first questionnaire aimed at identifying the psychosocial dimensions involved in the intention to do home-based physical activity; (b) reading persuasive messages aimed at promoting home-based physical activity and framed in different ways depending on the experimental condition; (c) filling out a second questionnaire aimed at detecting the evaluation of the messages received and any change in the intention to exercise at home.

We then developed a probabilistic graphical structure, i.e., a Dynamic Bayesian Network (DBN; Dagum et al., 1995; Murphy, 2012), as a first step in a process aimed at harnessing psychological models in the construction of automated interaction strategies via artificial intelligence. In doing this, we aimed at striking a balance between the *explanatory power* of the DBN, namely, its capacity of describing the causal connections among the psychological dimensions included in the theoretical model, and the *predictive capability* of the DBN, namely, its effectiveness in anticipating the effect of a specific interaction strategy. In other words, we aimed at achieving a good equilibrium between *what* can be predicted and *why* it can be predicted. The goal of achieving such a balance is relevant both for quantitative psychology (Yarkoni and Westfall, 2017) and for artificial intelligence (Adadi and Berrada, 2018).

To summarize, the main aim of our paper was to develop a probabilistic predictor in the form of a DBN, capable to explain and predict change in the intention to do physical activity at home after being exposed to messages on the subject. Such DBN is intended as the first step of an articulate process that has the ultimate goal of developing effective and automatic interaction strategies regarding behavior change.

In the rest of the paper, we first present the procedure and the measures employed in the empirical study, specifying the psychosocial theories we referred to in carrying it out. We then illustrate the main characteristics of the DBN, as structured predictor, and describe the methods adopted for its elicitation from the data collected in the study. The criteria to balance explanatory power and predictive capability, and the deterministic structure search of the DBN are also discussed. Then, in the Results section we illustrate the structure and parameters of the elicited DBN and its consistency with the psychosocial theoretical models. We finally discuss the advantages, limits, and future developments of our procedure, which will include a Deep Reinforcement Learning component for training a Deep Neural Network expected to drive online interactions with people.

## Methods

### Participants and Procedure

The present study was conducted following receipt of ethical approval by the Catholic University of the Sacred Heart (Milan). In April 2020, a sample of Italian participants was recruited to participate in a university study on the effects of public communication regarding the benefits of home-based physical activity. Participants were recruited by students of psychology courses at the Catholic University of Milan and received an email with a link to an online survey developed through the Qualtrics platform.

An initial sample of 280 participants accessed the online survey developed through the Qualtrics platform. First, participants completed a questionnaire measuring psychosocial dimensions involved in doing home-based physical activity (Time 1). Then, they were automatically and randomly assigned to four different experimental conditions, which consisted in being asked to read differently framed messages regarding the physical and psychological outcomes of exercising at home (Message Intervention). Finally, they were required to fill in a second questionnaire measuring their evaluation of the messages and again the psychosocial dimensions involved in home-based physical activity, to assess whether they had changed after message exposure (Time 2).

After excluding participants who either failed to pass the attention check questions in the questionnaires or did not complete them (*N* = 8), the final sample consisted of 272 participants (126 males, 142 females, 4 other; mean age = 42.97, *SD* = 14.98, age range = 18–70).

All data presented in this study can be found in the open repository at https://bitbucket.org/unipv_cvmlab/connecting_social_psychology_and_drl/.

### Theory-Based Measures

The theoretical starting point of our study was the integration of psychosocial models aimed at explaining behavior planning, its change through persuasive communication, and the matching effect between persuasive messages and recipients' characteristics (see also Di Massimo et al., 2019; Carfora et al., 2020a).

Regarding behavior planning, our reference model was the widely known Theory of Planned Behavior (TPB; Ajzen, 1991), according to which the *intention* to enact a certain behavior is predicted by the *attitude* toward the behavior (e.g., perceiving exercising at home as a useless activity), the *social norm* (e.g., feeling that others would approve of their regular exercising at home), and *perceived behavioral control* (e.g., being convinced to have internal and external resources to exercise at home). Over time, various researches have highlighted that the predictive capacity of TPB is further increased by the addition of two further dimensions, namely, *past behavior* (e.g., having exercised regularly in the past month) and *anticipated positive or negative emotions* concerning the outcome (e.g., anticipating that one will feel satisfied (or guilty) if one will (or will not) exercise at home).

Regarding the effects of persuasive communication, we referred to the Elaboration Likelihood Model (ELM; Petty and Cacioppo, 1986), according to which the long-term persuasiveness of a message largely depends on the *evaluation* and *systematic processing* of the message itself. Subsequent developments of this model have led to highlighting additional factors that can increase or vice versa decrease the persuasive effect of a message. Among the first, the perception of *trust* that the message arouses (Petty, 2018) and the positive *tone* of the message (Latimer et al., 2008a). Among the second, the perception of *threat* or *distress* activated by the message (Shen, 2015) and the negative *tone* of it (Latimer et al., 2008a).

Finally, in devising persuasive messages we referred to the Self-Regulatory Model of Message Framing (Cesario et al., 2013), according to which similar contents can be framed in different ways, for example by stressing either the positive or the negative outcomes of the recommended action. In a gain message the outcome of the action is formulated with a positive valence, whereas in a loss message the outcome is formulated with a negative valence. Gain messages can be further differentiated in messages describing an actual *gain* (e.g., “If you do home-based physical activity, you will improve your health”) and messages describing a *non-loss* (e.g., “If you do home-based physical activity, you will avoid damaging your health”). Similarly, loss messages can be further distinguished in messages describing an actual *loss* (e.g., “If you do not do home-based physical activity, you will damage your health”) and messages describing a *non-gain* (e.g., “If you do not do home-based physical activity, you will miss the opportunity to improve your health”).

Finally, previous research has shown that the persuasiveness of a message increases when its framing matches the recipient's regulatory focus (e.g., Yi and Baumgartner, 2009; Bertolotti et al., 2020). According to the Regulatory Focus Theory (RFT; Higgins, 1997), self-regulation with a *prevention focus* involves the avoidance of losses and the fulfillment of duties and obligations, while self-regulation with a *promotion focus* involves the pursuit of gains and the achievement of an ideal desirable state. Messages framed in terms of non-loss are more persuasive with people who have a prevalent focus of prevention, while messages framed in terms of gain are more persuasive with people who have a prevalent focus of promotion (Yi and Baumgartner, 2009). In this study we therefore introduced the regulatory focus measures at Time 1, to assess whether they would have an impact on intention change at Time 2, after exposure to differently framed messages.

#### Time 1 Measures

At the beginning of the survey, participants provided their informed consent and read the following statement: “We are interested in understanding what drives people to do physical activity at home in the absence of alternatives (i.e., in the impossibility of accessing parks, gyms, and open spaces). By physical activity at home we mean, for example: bodyweight workout (such as stretching, aerobics, push-ups, and abs), walking for at least 30 min (6,000 steps per day), training with weights and machines (such as stationary bikes and treadmills).” After that, participants answered to a series of questions measuring the relevant psychosocial dimensions investigated in the study.

*Prevention focus* was assessed using five items on a 7-point Likert scale adapted from the Health Regulatory Focus scale [e.g., “I often imagine myself being ill in the future… (1) Strongly disagree—(7) Strongly agree”; Ferrer et al., 2017]. The five items were used to compute a single prevention regulatory focus index, with higher values indicating a higher prevention focus. Cronbach's α was 0.87.

*Promotion focus* was assessed using five items on a 7-point Likert scale adapted from the Health Regulatory Focus scale [e.g., “I frequently imagine how I can achieve a state of “ideal health… Strongly disagree (1)—Strongly agree (7)”; Ferrer et al., 2017]. The five items were used to compute a single promotion regulatory focus index, with higher values indicating a higher promotion focus. Cronbach's α was 0.83.

*Past behavior*, related to physical activity *at home*, was assessed by asking how often participants engaged in exercising at home before the COVID-19 restrictions: “Before this period of restrictions, on average how many times a week did you exercise at home?. Never (1)—Every day (7).” Higher scores indicated a higher frequency of home-based physical activity before the COVID-19 restrictions.

*Past outdoor behavior*, related to *outdoor* physical activity, was assessed by asking how often participants engaged in exercising outside home before the COVID-19 restrictions: “Before this period of restrictions, on average how many times a week did you exercise outside home?. Never (1)—Every day (7).” Higher scores indicated a higher frequency of outdoor physical activity before the COVID-19 restrictions.

*Attitude* toward home-based physical activity was assessed using eight items on a semantic differential scale ranging from “1” to “7” (e.g., “I believe that doing physical exercises at home regularly is… useless—useful”; Caso et al., 2021). The eight items were used to compute a single attitude index, with higher values indicating a more positive attitude toward exercising at home. Cronbach's α was 0.93.

*Subjective norm* was assessed with three items using a Likert scale [e.g., “Most of the people important to me (partners, family, friends) think I should do physical exercises at home regularly… Strongly disagree (1)—Strongly agree (7)”; adapted from Carfora et al., 2020a,b]. The three items were used to compute a single subjective norm index, with higher scores indicating a higher level of it. Cronbach's α was 0.83.

*Perceived behavioral control* related to home-based physical activity was measured using five items on a seven-point Likert scale [e.g., “If I wanted, I would be able to do the physical activity regularly when I am feeling tired… (1) Strongly disagree—(7) Strongly agree”; adapted from Bandura, 1977]. The five items were used to compute a single index, with higher values indicating higher perceived behavioral control regarding exercising at home. Cronbach's α was 0.90.

*Anticipated positive emotions* for doing home-based physical activity were assessed with three items using a Likert scale [e.g., “If I do physical exercises at home regularly I will be satisfied… Strongly disagree (1)—Strongly agree (7)”; adapted from Carfora et al., 2018]. The three items were used to compute a single anticipated positive emotions index, with higher scores indicating a higher level of them. Cronbach's α was 0.92.

*Anticipated negative emotions* for not doing home-based physical activity were assessed with three items using a Likert scale [e.g., “If I do not do physical exercises at home regularly I will regret it… Strongly disagree (1)—Strongly agree (7)”; adapted from Carfora et al., 2018]. The three items were used to compute a single anticipated negative emotions index, with higher scores indicated a higher level of them. Cronbach's α was 0.89.

*Intention at Time 1* toward doing home-based physical activity was measured using three items on a seven-point Likert scale [e.g., “I intend to do physical exercises at home regularly in the next month… Strongly disagree (1)—Strongly agree (7)”; Clark and Bassett, 2014]. The three items were used to compute a single intention at Time 1 index. Higher scores indicated a greater intention to exercise at home at Time 1. Cronbach's α was 0.97.

**Figure 1:**
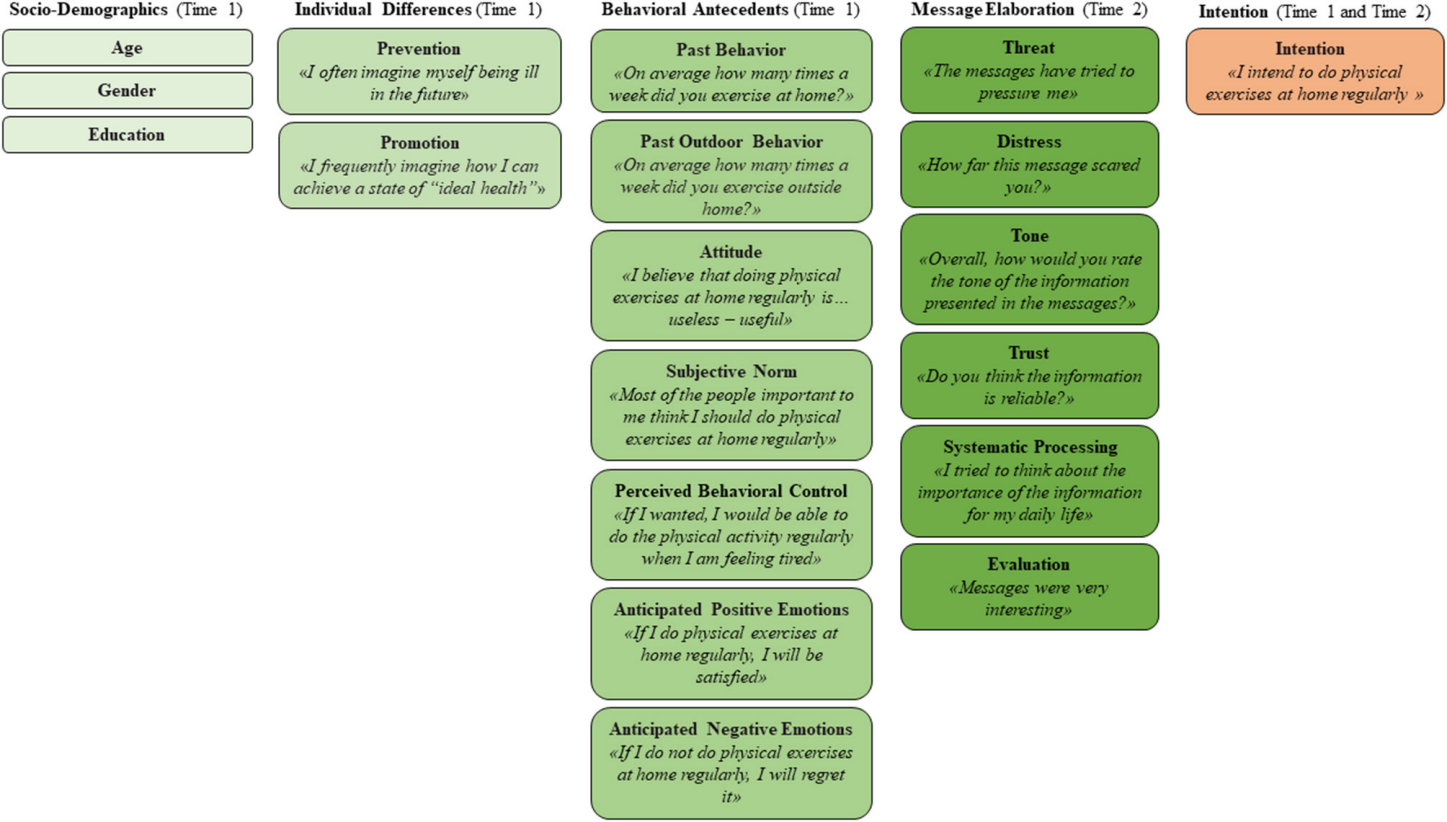
Psychosocial predictors of change in the intention to exercise at home, with examples of the measures employed.

A list of the above dimensions with examples of the items employed to measure them can be found in Figure 1.

#### Message Intervention

**Figure 2:**
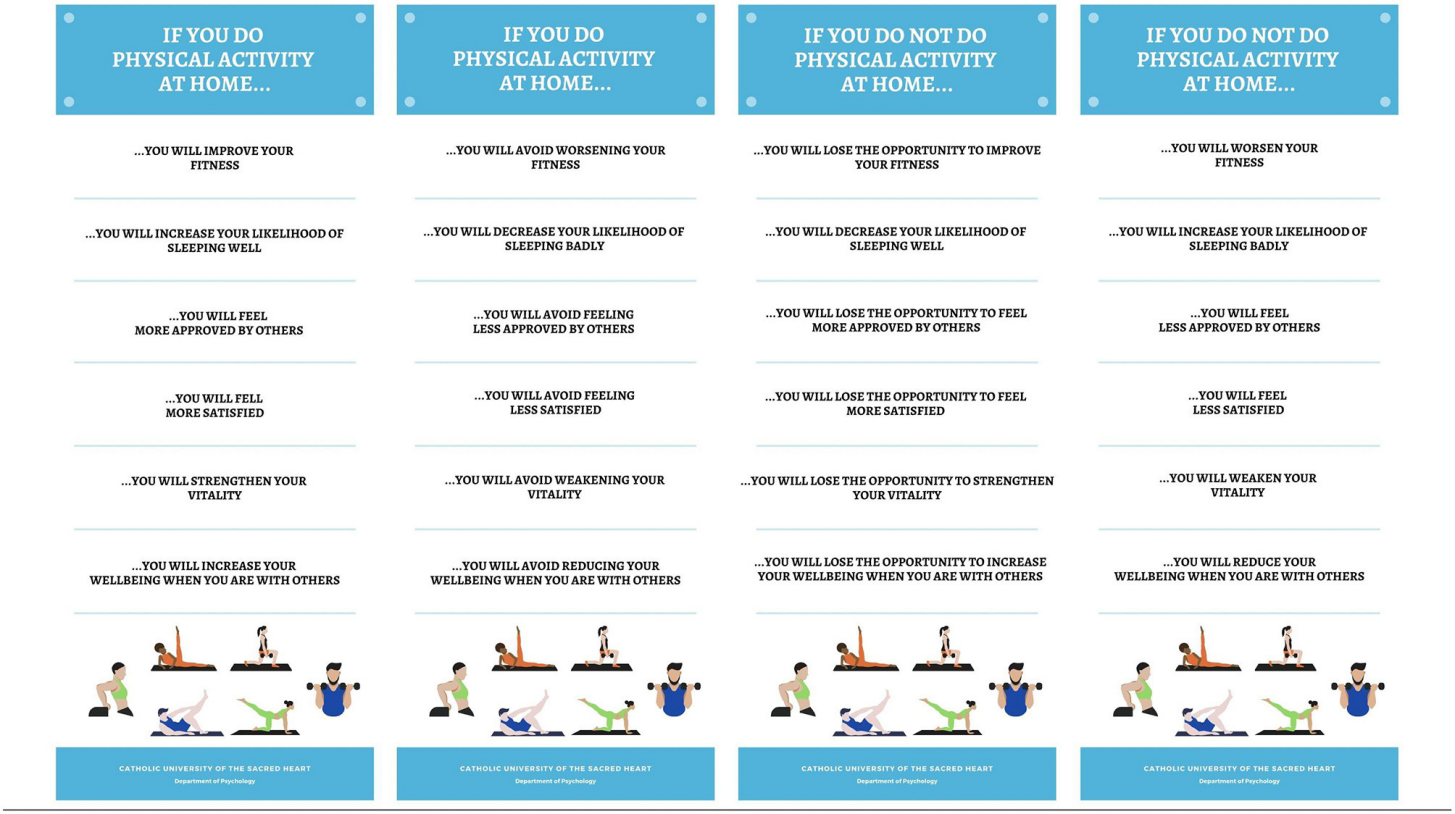
Infographics proposed in the gain, non-loss, non-gain, and loss message conditions.

After completing the first questionnaire, participants read an infographic with six messages describing the physical, psychological, and social consequences of doing home-based physical activity (Figure 2). All messages were formulated in prefactual terms (i.e., “If … then”; see Carfora and Catellani, 2021) and approximately consisted of 14 words each. Messages were formulated differently, according to the experimental condition to which participants had been randomly assigned. Participants in the *gain message condition* read messages emphasizing the positive consequences of doing home-based physical activity (e.g., “If you do physical activity at home, you will improve your fitness”). Participants in the *non-loss message condition* read messages informing how to avoid negative outcomes by doing home-based physical activity (e.g., “If you do physical activity at home, you will avoid worsening your fitness”). Participants in the *non-gain message condition* read messages emphasizing how doing home-based physical activity is associated with missing out positive consequences (e.g., “If you do not do physical activity at home, you will lose the chance to improve your fitness”). Finally, participants in the *loss message condition* read messages on the negative consequences of not doing home-based physical activity (e.g., “If you do not do physical activity at home, you will worsen your fitness”).

#### Time 2 Measures

After reading the messages, participants completed the second questionnaire, which measured the evaluation of the messages and once again the intention to exercise at home.

*Message-induced threat* was measured with four items on a 7-point Likert scale related to how much reading messages had made participants feel their freedom threatened [e.g., “The messages have tried to pressure me… (1) Strongly disagree – (7) Strongly agree”; adapted from Shen, 2015]. The four items were used to compute a single message-induced threat index, with higher values indicating higher perceived threat. Cronbach's α was 0.89.

*Message-induced distress* was assessed with five items on a 7-point Likert scale, pertaining to the degree to which reading messages induced distress [e.g., “How far this message scared you? … (1) Not at all – (7) Completely”; adapted from Brown and Smith, 2007]. All items were used to compute a single message-induced distress index, with higher values indicating higher distress after reading the messages. Cronbach's α was 0.86.

*Message tone* was measured with one item asking participants to rate the tone of the messages along the positivity-negativity dimension [“Overall, how would you rate the tone of the information presented in the messages? (1) Extremely negative – (7) Extremely positive”; adapted from Godinho et al., 2016]. Higher values indicated a more positive perception of the message tone.

*Message trust* was assessed with three items on a 7-point Likert scale [e.g., “Do you think the information presented in the message is reliable? (1) Not at all – (7) Extremely”; adapted from Godinho et al., 2016]. The three items were used to compute a single message trust index, with higher values indicating a higher trust in the messages. Cronbach's α was 0.92.

*Systematic processing* was measured with five items on a 7-point Likert scale, asking participants to state how deeply they had processed the information presented in the messages [e.g., “I tried to think about the importance of the information presented in the message for my daily life… (1) Strongly disagree – (7) Strongly agree”; adapted from Smerecnik et al., 2012]. The five items were used to compute a single systematic processing index, with higher values indicating a deeper processing of the messages. Cronbach's alpha was 0.91.

*Message evaluation* was assessed with six items on a 7-point Likert scale, regarding how participants evaluated the messages [e.g., “Messages were very interesting… (1) Strongly disagree – (7) Strongly agree”; adapted from Godinho et al., 2016]. The three items were used to compute a single message evaluation index, with higher values indicating a more positive evaluation of the messages. Cronbach's α was 0.92.

*Intention at Time 2* toward doing home-based physical activity was measured with the same three items employed at Time 1. Cronbach's α was 0.98.

*Intention change* was calculated subtracting the index *Intention at Time 1* from the index *Intention at Time 2*.

At the end of the questionnaire, participants reported their age, sex, and education.

A list of the above dimensions with examples of the items employed to measure them can be found in Figure 1.

### Dynamic Bayesian Network

We now describe the theoretical framework adopted for defining the probabilistic predictor (sections Learning Structure and Parameters From Data and Explanatory Power vs. Predictive Capability) and then describe the method used for eliciting the predictor from collected data (section Deterministic Structure Search).

A Bayesian Network B=(V,A,p) (BN, Darwiche, 2009) is a directed acyclic graph where nodes *V* correspond to the random variables in the model, *p* is a joint probability distribution over the set of random variables, and each link *A*⊆*V* × *V* represents an oriented dependence relation among two random variables. Together, nodes and directed arcs represent the structure of *p*, in terms of independence and conditional independence conditions among random variables. More precisely, assuming that {*X*_1_, …, *X*_*n*_} is the set of all random variables in the model, the joint probability distribution *p* can be factorized as

$$p\left(X_{1},\ldots ,X_{n}\right)=\prod_{i}p\left(X_{i}|\text{π}\left(X_{i}\right)\right)$$

where π(*X*_*i*_) is the set of *parents* of *X*_*i*_, i.e., the set of random variables whose representing nodes have an arc directed toward the node representing *X*_*i*_.

A *Dynamic* Bayesian Network (DBN; Dagum et al., 1995; Murphy, 2012) is a BN that also includes the representation of *time*, intended as a discrete sequence of instants. In a DBN:

- Each node is associated to a specific time instant.
- The same random variable may correspond to more than one node, at different times.
- All links must respect the orientation of time, either by connecting nodes at the same instant or by being oriented from a previous instant to a subsequent one.

**Figure 3:**
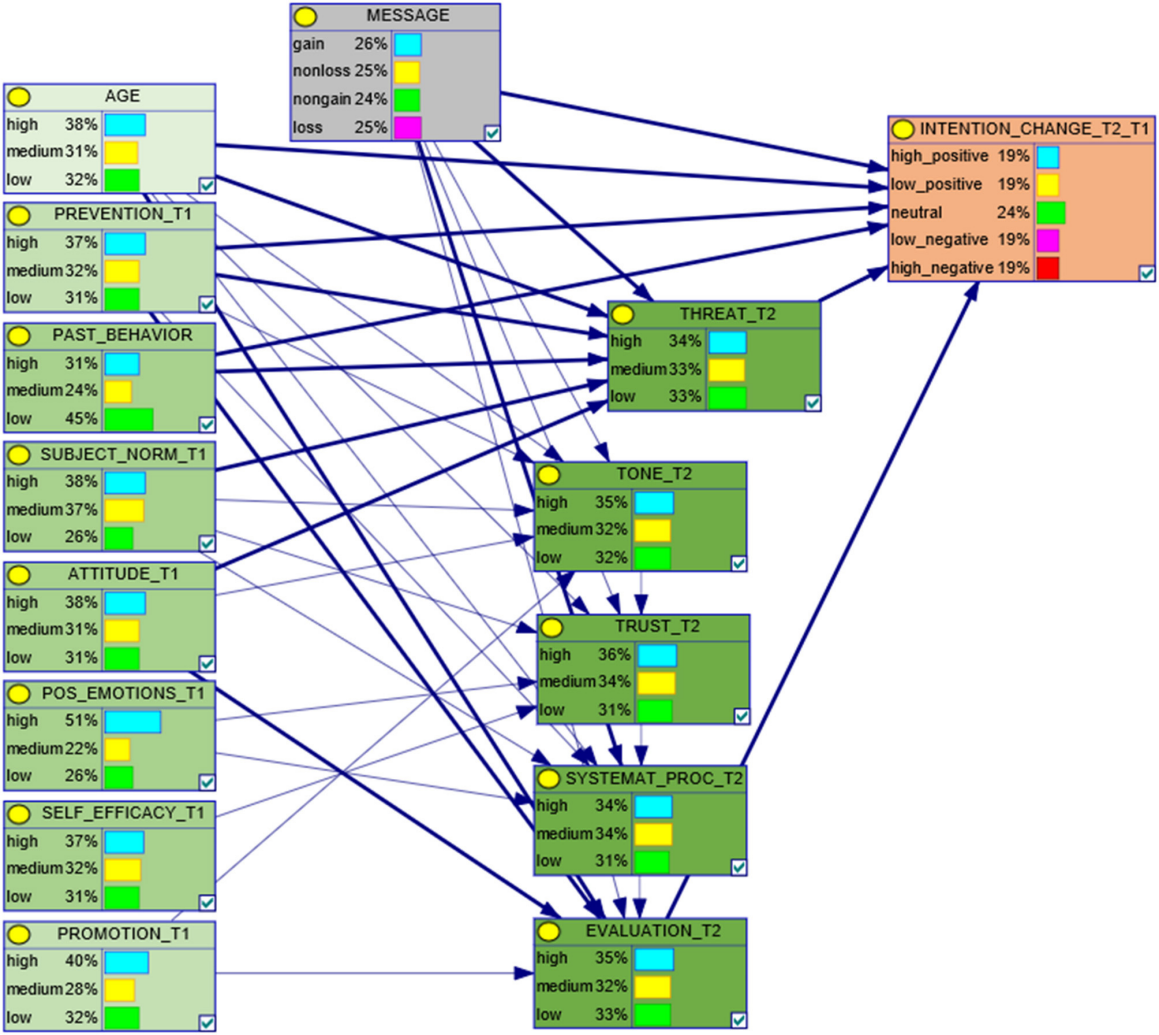
The elicited DBN structure.

As it can be seen in Figure 3, in our study the DBN was assumed to span across a sequence of three instants: Time 1, Message Intervention, and Time 2.

Being mean values of multi-item scales (Table 1), the indexes of the psychological dimensions calculated on the collected data can be assumed to be continuous. However, for computational simplicity, each corresponding random variable was assumed in this study to have values in the categorical scale {*low, medium, high*}, except for the target variable *Intention Change*, which was assumed to have values in the scale {*high-negative, low-negative, neutral, low-positive, high-positive*}. Indexes were discretized using *quantiles* (Nojavan et al., 2017): 20% quantiles for *Intention Change* and 33% quantiles for all the other variables.

**Table 1:** Means and standard deviations of the study measures.

**Time 1**			**Time 2**		
**Measure**	***M***	***SD***	**Measure**	***M***	***SD***
Prevention	3.68	1.39	Message-induced threat	5.79	1.52
Promotion	5.35	0.91	Message-induced distress	4.92	1.17
Past behavior	2.63	1.89	Message tone	5.19	1.29
Past outdoor behavior	4.39	1.76	Message trust	5.47	0.98
Attitude	1.21	0.43	Systematic processing	4.97	1.24
Perceived behavioral control	4.92	1.17	Message evaluation	4.60	1.24
Subjective norm	5.19	1.25	Intention	5.17	1.70
Anticipated positive emotions	5.43	1.46			
Anticipated negative emotions	4.36	1.76			
Intention	5.15	1.75			

#### Learning Structure and Parameters From Data

In general, once the structure of a DBN has been defined, the probability distribution *p* can be learned from experimental data, in a direct form. The learning process is an optimization aiming to compute the *maximum likelihood estimator* (MLE):

$$\theta_{MLE}:\text{​ }=\underset{\theta }{\text{argmax}}\;L\left(\theta ,D\right)$$

where *θ* is the set of probability values, *D* are the collected data and *L* is the likelihood function. Omitting details, in the case of discrete Bayesian Networks the above optimization process could be solved analytically, by computing all required probabilities as frequency ratios in *D* (Murphy, 2012). However, such direct method is rarely used since it is vulnerable to missing data, a circumstance that occurs very often with limited datasets. In practice, other methods such as the EM algorithm (Dempster et al., 1977) are preferred since they are more robust and can deal with missing data.

A more complicate task, which has been subject to intense research, is eliciting from data the structure of the Bayesian Network (i.e., the acyclic graph) that best synthesizes the information collected in the experiments. In many commonly adopted approaches, a scoring function is used to evaluate candidate structures (Koller and Friedman, 2009). An obvious choice for this would be the likelihood function itself. One problem in doing so, however, is that the likelihood function is monotonically increasing with the number of nodes and arcs in the network. In other words, a Bayesian Network including one node per each measured variable and being a fully connected (acyclic) graph is due to attain the maximal likelihood in all cases. To counter this tendency, the *Bayesian Information Criterion* (BIC) includes another term that measures the complexity of the network:

$$\begin{array}{l} BIC\left(B,D\right):=l\left(\theta ,D\right)-\frac{\log N}{2}|B| \end{array}$$

where B is the Bayesian Network, *l*(*θ*, *D*): = log*L*(*θ*, *D*) is the log-likelihood, *N* is the size of the dataset and |B| measures the number of nodes and arcs in the graph. The second term above is also called *description length*. In our work, however, we preferred a still different way to counter the tendency to structure growth induced by functions as the likelihood, as it will be explained in section Deterministic Structure Search.

Once a scoring function has been chosen, the subsequent step is defining a procedure for finding the graph structure of B that maximizes the given score. Unfortunately, this problem is NP-hard (Koller and Friedman, 2009) in general and therefore impervious to exhaustive search in almost all practical cases. Several heuristic search strategies have been proposed in the literature to circumvent this problem (e.g., see Cheng et al., 2002). In most cases, however, these strategies are stochastic, since they imply random choices of some sort (Scanagatta et al., 2019). In our study, we preferred adopting a more problem-specific and deterministic search strategy together with a suitable scoring function, as it will be explained in section Deterministic Structure Search.

#### Explanatory Power vs. Predictive Capability

Given the stated purposes, our objective was to achieve a DBN that could predict the value of the target variable *Intention Change* (whose index was computed subtracting *Intention* at Time 1 to *Intention* at Time 2) relying only on Time 1 observations and Message Intervention. In other words, the objective was estimating the conditional probability:

p(target variable | Time 1 observations, Message Intervention)

for all message types considered. One possible way of evaluating the effectiveness of a categorical predictor of this kind is through *accuracy*. Calling *X*_*t*_ the target variable, for conciseness, the value predicted by the DBN will be:

$$v_{pred}:\text{​ }=\underset{v}{\text{argmax}}\;p\left(X_{t}=v\;|\;Obs,\;Msg\right)$$

where *v* is one of the categorical values of *X*_*t*_ and *p* is the probability computed by the DBN. Accuracy is computed by considering each participant in the data collection, computing the probability of each value *v* given Time 1 observations and the Message Intervention that has been delivered to the participant in point. Accuracy is defined as the ratio of how many times we succeed in having:

$$\begin{array}{l} v_{pred}=v_{true} \end{array}$$

where *v*_*true*_ is the value actually observed, over the size *N* of the dataset.

Given our objectives, the effectiveness of the DBN was intended as a balance between maintaining a clear connection with the theoretical background of reference and the generalization capability of predicting the target index for unseen subjects, given limited observations. In this perspective, accuracy could be evaluated both in-sample, for data explanation, and out-of-sample, to assess the predictive power of a DBN. In-sample accuracy can be evaluated by first learning the DBN parameters from the entire dataset, as described in section Learning Structure and Parameters From Data, and then predicting the target index in each record individually, in the same dataset, using partial observations only. Out-of-sample accuracy can be estimated via the *k-fold cross-validation* method (Allen, 1974). In our case, however, we preferred the *leave-one-out* method (Raschka, 2018): one participant *d* is removed from the dataset *D*, then probabilities *θ* are learnt from (*D*−*d*) and accuracy is tested for *d*. The procedure is repeated for all participants in *D* and the resulting success ratio is computed.

**Figure 4:**
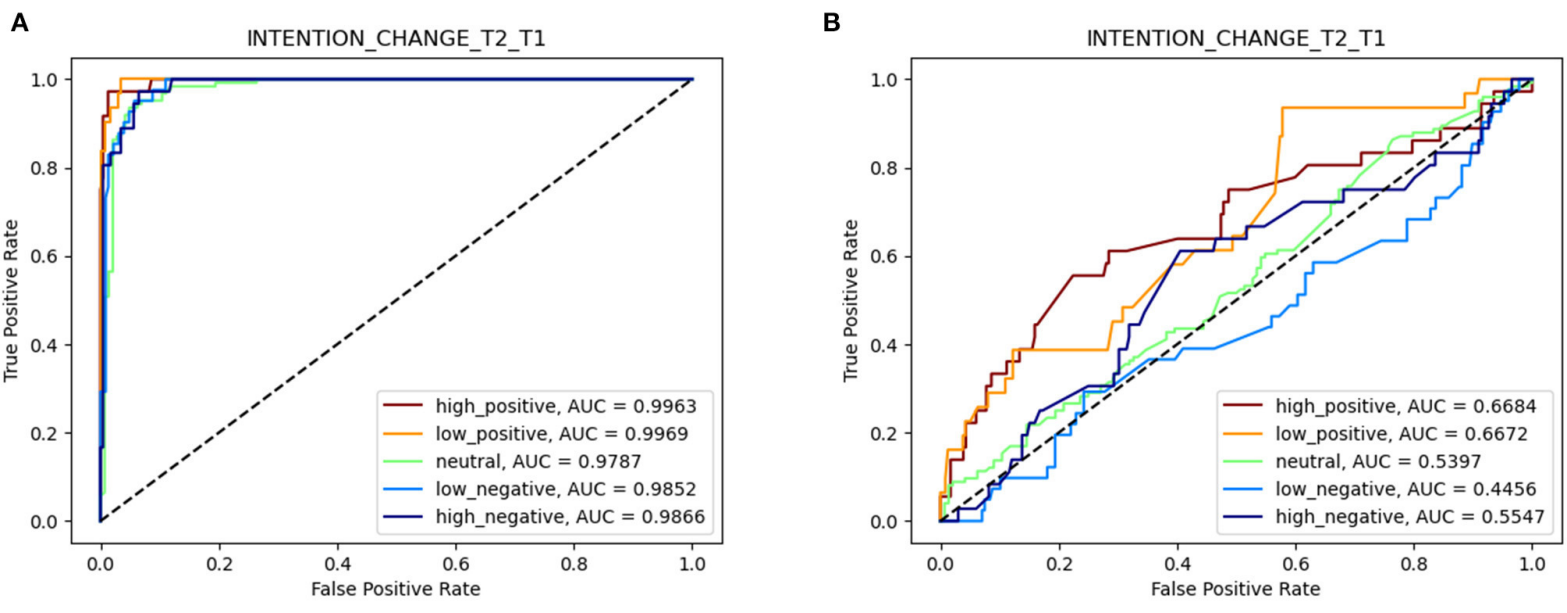
Multivalued ROC curves obtained for the elicited DBN. elicited DBN. **(A)** In-sample ROCs. **(B)** Out-of-sample ROCs.

Accuracy, however, is a somewhat crude measure in that it considers only the highest probability value, conditioned on known information, and not the entire distribution. A better metrics is *Area Under Curve* (AUC; Fawcett, 2006) which measures the area under the curve traced by points:

(p(FP|γ),p(TP|γ))

where *FP* and *TP* are *False Positive* and *True Positive* value assignments, respectively, obtained when accepting a predicted value *v* whenever *p*(*X*_*t*_ = *v*)≥γ, and γ varies in [0, 1]. Such curve is also called *Received Operating Characteristic* (*ROC*). Examples of ROC curves are shown in Figure 4. Given that the target variable in our case had five categorical values, in the present study the multiclass version of AUC (i.e., mAUC–Hand and Till, 2001) was used.

In summary, in our study we computed the mAUC values for both in-sample and out-of-sample (i.e., through leave-one-out) validation and we considered the average of the two as our main scoring function for selecting the best possible structure of the DBN.

#### Deterministic Structure Search

Despite its advantages, computing the mAUC is expensive (in particular for the leave-one-out validation) and this does not match well with the complexity of structure searching. This raises the need to pre-select candidate structures using a more conveniently computable scoring function.

In this perspective, as shown by Koller and Friedman (2009), the log-likelihood function can be expressed as:

$$\begin{array}{l} l\left(\theta ,D\right)=\text{N}\left(\underset{i}{\sum }IG\left(X_{i};\pi \left(X_{i}\right)\right)-\underset{i}{\sum }H\left(X_{i}\right)\right) \end{array}$$

where *N* is the size of the dataset, *H* is the *entropy*:

$$\begin{array}{l} H\left(X\right):=-\underset{X}{\sum }p\left(X\right)\log p\left(X\right) \end{array}$$

and *IG* is the *information gain* (Jiang et al., 2015):

$$\begin{array}{l} IG\left(X;Y_{1},\ldots ,Y_{n}\right):=H\left(X|Y_{1},\ldots ,Y_{n}\right)-H\left(X\right) \end{array}$$

where the *conditional entropy* is defined as:

$$\begin{array}{l} H\left(X|Y_{1},\ldots ,Y_{n}\right):=-\underset{X,Y_{1},\ldots ,Y_{n}}{\sum }p\left(X,Y_{1},\ldots ,Y_{n}\right)\\ \;\log\frac{p\left(X,Y_{1},\ldots ,Y_{n}\right)}{p\left(Y_{1},\ldots ,Y_{n}\right)} \end{array}$$

In all the above equations, *p* can be construed as the empirical probability distribution, estimated as frequency ratios in the dataset.

In other terms, in the above decomposition the log-likelihood score is shown to be proportional to information gain of the conditional probabilities in B minus a constant entropy term, i.e., which does not depend on the structure of B. Furthermore, information gain values are terms in a sum and could be optimized separately, within the limit of not introducing cyclic dependencies in the graph.

In the light of the above, in our study we used information gain as a preliminary scoring function, to select the most promising structures. We then computed the combined mAUC metrics (i.e., in-sample and out-of-sample) of the later structures, to select the most effective one. Our procedure was as follow:

1. We first considered the target variable *X*_*t*_ and we computed the information gain for all possible subsets of parents of size in between 2 and 8, chosen among all other random variables (i.e., Time 1, message intervention, Time 2).
2. Having selected the best subsets of parents for *X*_*t*_, one per each size in the above range, we expanded each Time 2 variable in each selected parenthood by measuring the information gain of all possible subsets of size in between 2 and 8, chosen among the remaining variables, avoiding cycles.
3. For each combination of sizes (i.e., one for the parenthood *X*_*t*_ and one for the parenthood of each Time 2 node), we pre-selected one structure, namely the one with the largest overall information gain, hence the highest likelihood.
4. For all the pre-selected structures we computed the combined in-sample and out-of-sample mAUC metrics, to select the most effective one.

Note that step 2 above was completed when all Time 2 nodes became expanded, so that all of them had a parenthood rooted in Time 1 nodes, either directly or indirectly. The need to do so derived from the objective of achieving a predictor of the target variable *Intention Change* that relies on Time 1 observations only.

To avoid a combinatorial explosion in the number of candidate structures, in the above procedures all parenthoods of Time 2 nodes in each structure were imposed to have the same size. For instance, in the structure that resulted as best in its combination of ranges (see Figure 3) all Time 2 nodes have 6 parents exactly. Clearly, this entails the risk of a certain redundancy in the structures produced. To evaluate this aspect, for all selected structures, we also computed the *interaction strength* (Zeng et al., 2016) on each set of parents:

$$\begin{array}{l} IS\left(X;Y_{1},\ldots ,Y_{n}\right):=IG\left(X;Y_{1},\ldots ,Y_{n}\right)-\underset{i}{\sum }IG\left(X;Y_{i}\right) \end{array}$$

Interaction strength measures the difference between the cumulative information gain of a subset of parents for a given variable over the sum of each individual information gains in the same subset. Unlike information gain, interaction strength is not monotonically increasing with the number of variables but has a peak that is expected to correspond to the strongest interacting parenthood. In our case, interaction strength was computed, for the selected structures, for all possible combinations of parents among the ones selected through the procedure described above.

The relevant advantage of the above chosen method is that the structure selection procedure is entirely deterministic and repeatable. The theoretical aspects of psychosocial models play a crucial role in the initial phase of dimensions and measures selection, whereas their interrelations are hypothesized only implicitly. Subsequently, starting from the analysis of the experimental data, structure and parameters of the probabilistic predictor are learned in an automatic way, by assuming the target variable *Intention Change* and the temporal sequence of events as the only constraints. The results thus obtained are in keeping with the implicit theoretical assumptions and this adds credibility to the proposed procedure.

## Results

The DBN structure described in Figure 3 resulted as the best one among those generated via the procedure described in section Deterministic Structure Search, applied to the dataset of experimental measures. Figure 4 describes the multivalued ROC curves obtained for the DBN in Figure 3, with in-sample and out-of-sample tests, respectively. The latter test was performed with the leave-one-out technique. In these tests, the DBN in point scored a combined mAUC value of 0.783 (with in-sample and out-of-sample values of 0.989 and 0.577, respectively).

As anticipated in the previous section, all parenthoods in the DBN were tested for interaction strength. The strongest interaction subsets in each parenthood are shown by thicker arrows in Figure 3. As it could be expected, the parenthood of the target variable *Intention Change* resulted as coincident with the strongest interacting subset. The same resulted for variable *Threat*. On the other hand, the strongest interacting subset for variable *Evaluation* included just 3 of 6 parents. Time 2 variables *Tone, Trust*, and *Systematic Processing* could not be found among the strongest interacting parenthoods.

Interestingly however, although to a minor extent, even marginal interactions were proven to have a role in determining the overall performance of the DBN in point. In fact, the reduced DBN structure obtained by considering only the thicker arrows in Figure 3 and by discarding unconnected nodes, scored a combined mAUC value of 0.762 (0.960, 0.565). This result is also representative of the fact that, in our case, interaction strength did not prove to be as effective as the information gain for the pre-selection of candidate DBN structures.

For the results presented, the action of learning DBN parameters was performed, for both in-sample and out-of-sample tests, via the EM algorithm as implemented in the SMILE library, by BayesFusion^1^. All other computations were performed with custom code, made with Python and Numpy^2^. The complete definition of the DBN structure described in Figure 3 can be found in the same open repository mentioned in section Participants and Procedure.

## Discussion

As part of an interdisciplinary project between social psychology and artificial intelligence, in this paper we presented a deterministic method for the elicitation of a DBN, starting from data on the psychosocial antecedents of the intention to exercise at home and intention change after being exposed to persuasive messages on the issue. This method constitutes a first step toward the development of deep reinforcement learning techniques which will allow devising personalized interaction strategies based on consolidated psychosocial models of behavior change. In this discussion, we will first focus on the theoretical consistency of the elicited DBN and we will then describe its strengths and limits.

### Theoretical Consistency of the Elicited DBN

The DBN structure that emerged from the analysis turned out to be largely consistent with the psychosocial literature of reference. It also highlighted the presence of interesting relationships between measures related to the different psychosocial theories we referred to when devising our integrated model. We will now illustrate the DBN structure analyzing the strongest links between the variables and interpreting them in the light of the psychosocial theories we referred to when selecting the variables to be included in the initial model.

We start by examining the direct predictors of *Intention Change*, i.e., change in the intention to exercise at home after reading the messages. Message framing directly predicted *Intention Change*, suggesting that the four different message frames employed in the study affected differently the observed changes in the behavioral intention of the recipients. Message-induced threat also had a direct impact on *Intention Change* and was in turn directly influenced by message framing. Therefore, different message frames triggered different levels of perceived threat in the recipients, which in turn influenced the change in the intention to exercise at home. This finding is consistent with previous research in the domain of the effects of communication on health. According to the psychological reactance theory, when individuals feel that a health message is prompting them to accept a certain behavior, they may not process it accurately and instead respond defensively, downplaying its recommendation and not changing their intention (Liberman and Chaiken, 1992; Falk et al., 2015; Howe and Krosnick, 2017). According to the theory of self-affirmation (Steele, 1988; Sherman and Cohen, 2006), this defensive reaction against threatening messages is based on the attempt to maintain the perception of being able to control the relevant results. When this defensive mechanism is activated, people can attempt to protect it by rejecting such threatening information (e.g., Strachan et al., 2020).

Message evaluation also had a direct influence on *Intention Change* and was directly influenced by message framing. Message evaluation was also influenced, albeit less strongly, by the systematic processing of the message, which in turn was influenced by trust in the message and the perceived positive or negative tone of the message itself. This chain of influences is consistent with previous literature on persuasive communication showing that intention changes depend upon the likelihood of a persuasive message being positively evaluated by the receiver (Petty and Cacioppo, 1986; Eagly and Chaiken, 1993). The positive evaluation of a message, in turn, depends on systematic processing (Chaiken, 1980), which implies cognitive effort in considering the content of a message. Previous literature also showed that people tend to evaluate the trustworthiness of a message before processing it (Schlegelmilch and Pollach, 2005). Finally, trust in a message is influenced by how receivers perceive its tone. A negative tone can more easily be perceived as an open persuasive attempt and can therefore induce lower trust toward the message (Yalch and Dempsey, 1978).

*Intention Change* was directly predicted not only by message framing and message-related variables, but also by three variables measured at Time 1, namely, participants' age, frequency of past exercising at home, and prevention focus. Besides having a direct impact on *Intention Change*, participants' age had an indirect impact on it, through the mediation of message-induced threat and message evaluation. These results are consistent with a vast amount of past studies showing the effect of age on physical activity over lifespan (Varma et al., 2017), also during the COVID-19 pandemic (Alomari et al., 2020). Unlike age, gender and education did not have either a direct or indirect effect on *Intention Change*. This result is consistent with McCarthy et al. (2021), who found that socioeconomic group and gender were not associated with changes in physical activity during the COVID-19 restrictions. As to the frequency of past home exercising, it predicted *Intention Change* both directly and via the mediation of message-induced threat. This finding is strongly supported by past research, which offers wide evidence that past behavior is one of the largest contributors to the explanation of physical activity (Young et al., 2014). It is worth noting that the frequency of physical exercise outside home (which was also part of the initial model) did not enter in the final DBN and therefore did not turn out to be among the main predictors of *Intention Change*. This result may be explained by the fact that people do not perceive physical activity at home as equivalent to physical activity outside home, and therefore this latter activity may not play a significant role in predicting a change in the intention to train at home.

Prevention focus also directly predicted a change in the behavioral intention. It had both a direct influence on *Intention Change* and an indirect influence, via the mediation of message-induced threat and message evaluation. Avoidance of losses and the fulfillment of duties and obligations evidently influenced a change in recipients' intention after being exposed to differently framed messages fostering exercise at home. This result is consistent with previous research showing that the effect of differently framed messages may vary according to the recipient's regulatory focus (Latimer et al., 2008b; Pfeffer, 2013). In our study, the promotion focus also had a link, albeit only an indirect one, with *Intention Change*. However, it was a weaker link than the one of the prevention focus, mediated only by the evaluation of the message and not also by the threat induced by the message, as was the case with the prevention focus. Understanding why prevention focus had more impact on *Intention Change* than promotion focus would require analyses that go beyond the ones presented in this paper. For example, it may be the case that individuals with a high promotion focus are basically more oriented to do physical activity than individuals with a high prevention focus, to achieve an ideal of well-being and health. If so, their intention to do physical activity may be already high and therefore they would be less likely to be persuaded to further enhance this activity by messages focused on the issue.

As to the extended TPB variables measured at Time 1 (past behavior, attitude, subjective norm, perceived behavioral control, and anticipated emotions), as discussed above only past behavior had a direct impact on *Intention Change*. Attitude and subjective norm also had an influence on *Intention Change*, but this influence was mediated by message-related variables. Attitude had an influence on *Intention Change* via the mediation of message-induced threat and message evaluation. This result is consistent with previous studies on the influence of attitudes and message framing on intention change in health-related domains (e.g., Carfora and Catellani, 2021; Caso et al., 2021). Subjective norm had an impact on *Intention Change* via the mediation of message-induced threat. Previous research showed that subjective norm may exert its influence on intention through perceived threat (Becker and Maiman, 1975). Consistently, we can hypothesize that when people attach importance to the recommendations and expectations of others, they may tend to feel more threatened by the risks presented in persuasive messages. A confirmation of this link would, however, deserve further empirical support.

Overall, the DBN structure that emerged from our analysis was largely consistent with the psychosocial literature in the area. At the same time, it contributed to enrich it, showing the presence of interesting and plausible links between variables belonging to the three different psychosocial theories that we took as a reference when constructing the initial model.

### Methodological Strengths of the Elicited DBN

The approach we followed in the elicitation of the DBN has several methodological strengths which can be traced back to three main points.

First, in our method the structure selection procedure was entirely deterministic and repeatable and nevertheless, as discussed above, led to a structure which was theoretically consistent. Notably, the adoption of the discretization of the values of the psychosocial measures on the one hand necessarily introduced approximations, but on the other hand simplified data analysis and allowed the identification of a significant structure from a small sample.

Second, the intention of balancing explanatory power with predictive capability led us to adopting a selection metric for eliciting the DBN which, albeit at the cost of increased computation complexity, effectively counteracted the tendency of the common likelihood metrics to reward the most complex structures. In this way, we believe it is also possible to prevent the overfitting, intended as the result of overestimating in-sample over out-of-sample performances, of structural models with respect to the sample of collected data (Yarkoni and Westfall, 2017). As a matter of fact, the in-sample and out-of-sample performances of the elicited DBN were divergent in the measured values (see Figure 4). Nevertheless, it is reasonable to expect that such gap could significantly decrease whenever the size and relevance of the sample could be made to increase.

Third, the DBN obtained was effective from both an explanatory and predictive point of view. In particular, the structure of the DBN was easy to interpret and relate to the psychological models that were assumed as the starting point. Its efficacy is a first important step for the creation of an artificial intelligence system that will translate the results of psychological research into automatic interaction and interventions policies for improving many people's lives. Once fully operational, these systems will require less time and economic efforts to be operated, compared to those required by putting the same psychological models at work through human intervention alone.

### Limits

Our research has some limitations, related to the quality of the data collected, data analysis and the development of the DBN. As for the data, these were collected on a non-representative sample of the population and with reference to the intention to carry out physical activity at home in a very particular historical moment, that of the first wave of the Covid-19 pandemic. This makes it difficult to extend our results to different populations and times. Furthermore, it should be noted that the measurement of the effectiveness of the messaging interventions employed was based on the change in the intention to carry out physical activity at home and not on measures relating to the actual performance of this activity, such as those that may be offered by bracelets or wearable sensors worn by participants. Regarding the intention measurement, we used a Likert scale that measured the participants' agreement with intending to do physical exercises at home. Future scale should instead use probability scales to reduce the likelihood of response-style biases (Morwitz and Munz, 2021).

As for data analysis and learning of structure and parameters of the DBN, the reduced size of data sample was definitely a limiting factor, as it can be observed in the divergence between in-sample and out-of-sample performances (see Figure 4). Therefore, the actual effectiveness of the predictor obtained should be further tested in a real-world application scenario.

### Future Developments

The method for DBN elicitation described in this paper constitutes the first part of an articulated path. This same method is currently being tested within a purpose-specific framework based on Deep Reinforcement Learning (DRL; François-Lavet et al., 2018; Sutton and Barto, 2018) to train a Deep Neural Network component, which is intended to drive online interactions with actual people, by applying the psychosocial principles described.

Further on, the DRL software framework under construction is expected to evolve to include the capability to collect additional experience and allow the incremental improvement of the DBN itself. In this perspective, the DBN is intended to play a fundamental role, in guaranteeing the explainability of the behavior of the AI system, giving to both psychologists and experts of artificial intelligence the power to monitor and intervene in the learning procedure.

Thanks to the application of DRL techniques it will be possible to calculate the utility deriving from sending messages with different framing to people who differ from each other as regards the psychosocial dimensions underlying the behavior under study.

## Conclusion

In conclusion, our results show that social psychology and artificial intelligence can usefully interact to develop automatic interaction strategies aimed at supporting behavior change in the direction of well-being. As we have seen, this interaction helps overcoming some of the constraints the two disciplines often encounter when developing models that are expected to find application in real life. The possibilities of applying a methodology such as the one tested here are many and concern various areas, virtually all those in which it is reasonable to think that sending personalized messages to the recipient through automatic systems can have positive effects for the well-being of the person. Much can therefore be done thanks to the integration of social psychology and artificial intelligence, moving from the assumptions that the wealth of processing and production of new data allowed by artificial intelligence systems can ultimately be a way to enrich and improve the experience of people, for whom artificial intelligence systems have reason to be.

## Data Availability Statement

All data presented in this study can be found in the open repository at https://bitbucket.org/unipv_cvmlab/connecting_social_psychology_and_drl/.

## Ethics Statement

The studies involving human participants were reviewed and approved by Ethics Committee of the Catholic University of the Sacred Heart. The patients/participants provided their written informed consent to participate in this study.

## Author Contributions

PC proposed the research questions, planned the research design, and took responsibility for the manuscript. She also thoroughly revised the manuscript with regard to content and style. VC supervised data collection and analysis and participated in the interpretation of the results. MP designed the elicitation procedure for the probabilistic predictor, implemented the code, and carried out the computational experiments. All authors contributed to the article and approved the submitted version.

## Conflict of Interest

The authors declare that this study received funding from Athics s.r.l. The funder was not involved in the study design, collection, analysis, interpretation of data, the writing of this article, or the decision to submit it for publication.

## Footnotes

^1^See https://www.bayesfusion.com/

^2^See https://numpy.org/
